# Matching positive end-expiratory pressure to intra-abdominal pressure improves oxygenation in a porcine sick lung model of intra-abdominal hypertension

**DOI:** 10.1186/cc11840

**Published:** 2012-10-26

**Authors:** Adrian Regli, Rohan Mahendran, Edward T Fysh, Brigit Roberts, Bill Noffsinger, Bart L De Keulenaer, Bhajan Singh, Peter V van Heerden

**Affiliations:** 1 Intensive Care Unit, Fremantle Hospital, Alma Street, Fremantle 6160, Australia; 2 School of Medicine and Pharmacology, The University of Western Australia, 35 Stirling Highway, 6009 Crawley, Australia; 3 Medical School, The University of Notre Dame Australia, 19 Mouat Street, 6959 Fremantle, Australia; 4 Intensive Care Unit, Sir Charles Gairdner Hospital, Hospital Avenue, 6009 Nedlands, Australia; 5 Department of Pulmonary Physiology and Sleep Medicine, Sir Charles Gairdner Hospital, Hospital Avenue, 6009 Nedlands, Australia; 6 Medical Intensive Care Unit, Hadassah University Hospital, Ein Kerem, 91120 Jerusalem, Israel

## Abstract

**Introduction:**

Intra-abdominal hypertension (IAH) causes atelectasis, reduces lung volumes and increases respiratory system elastance. Positive end-expiratory pressure (PEEP) in the setting of IAH and healthy lungs improves lung volumes but not oxygenation. However, critically ill patients with IAH often suffer from acute lung injury (ALI). This study, therefore, examined the respiratory and cardiac effects of positive end-expiratory pressure in an animal model of IAH, with sick lungs.

**Methods:**

Nine pigs were anesthetized and ventilated (48 +/- 6 kg). Lung injury was induced with oleic acid. Three levels of intra-abdominal pressure (baseline, 18, and 22 mmHg) were randomly generated. At each level of intra-abdominal pressure, three levels of PEEP were randomly applied: baseline (5 cmH_2_O), moderate (0.5 × intra-abdominal pressure), and high (1.0 × intra-abdominal pressure). We measured end-expiratory lung volumes, arterial oxygen levels, respiratory mechanics, and cardiac output 10 minutes after each new IAP and PEEP setting.

**Results:**

At baseline PEEP, IAH (22 mmHg) decreased oxygen levels (-55%, *P *<0.001) and end-expiratory lung volumes (-45%, *P *= 0.007). At IAP of 22 mmHg, moderate and high PEEP increased oxygen levels (+60%, *P *= 0.04 and +162%, *P *<0.001) and end-expiratory lung volume (+44%, *P *= 0.02 and +279%, *P *<0.001) and high PEEP reduced cardiac output (-30%, *P *= 0.04). Shunt and dead-space fraction inversely correlated with oxygen levels and end-expiratory lung volumes. In the presence of IAH, lung, chest wall and respiratory system elastance increased. Subsequently, PEEP decreased respiratory system elastance by decreasing chest wall elastance.

**Conclusions:**

In a porcine sick lung model of IAH, PEEP matched to intra-abdominal pressure led to increased lung volumes and oxygenation and decreased chest wall elastance shunt and dead-space fraction. High PEEP decreased cardiac output. The study shows that lung injury influences the effects of IAH and PEEP on oxygenation and respiratory mechanics. Our findings support the application of PEEP in the setting of acute lung injury and IAH.

## Introduction

Intra-abdominal hypertension (IAH) is defined as a sustained intra-abdominal pressure (IAP) above or equal to 12 mmHg [1]. IAH is present in 30% to 60% of critically ill patients [2-4] and mortality increases in proportion to the degree of IAH [3]. IAH is associated with reduced cardiac output by an increase in systemic vascular resistance and a decrease in venous return [5,6]. The raised abdominal pressures, together with the impaired cardiac output reduce blood flow to vital intra-abdominal organs, such as kidneys and liver [5,6].

IAH is also associated with atelectasis and impaired lung function, resulting from a cephalad shift of the diaphragm [7-10]. IAH has been reported to reduce lung volumes and increase trans-diaphragmatic pressures, inspiratory airway pressures and chest wall elastance [8,10-13].

IAH appears to reduce oxygenation only minimally in the presence of healthy lungs [14], which has been attributed to a redistribution of blood flow from atelectatic dorsal to better ventilated ventral lung regions and thereby only minimally affecting ventilation-perfusion matching [14]. However, in injured lungs IAH can substantially impair oxygenation [8,15] that is probably due to an increase in pulmonary edema [8].

The optimal level of positive end-expiratory pressure (PEEP) in the setting of IAH is controversial. While increased levels of PEEP have been suggested to improve lung function [9], this approach carries the risk of regional pulmonary overdistension injury [11,16] and hemodynamic compromise [6].

Two clinical trials have assessed the effect of different PEEP levels on respiratory function in patients with IAH and acute lung injury (ALI) or acute respiratory distress syndrome (ARDS) with conflicting results [12,17].

The aim of this experimental study was to examine the effect of different PEEP levels on oxygenation and respiratory mechanics in the setting of IAH and lung injury. We hypothesized that PEEP would attenuate the IAH-induced decline in gas exchange in a porcine sick lung model with IAH.

We tested two different PEEP levels that were adjusted to the degree of IAP to counter-balance the trans-diaphragmatic pressures as previously suggested [9]. Furthermore, we previously were able to show that the application of higher than usual PEEP levels that were adapted to the degree of IAP was able to reverse lung volumes in a healthy porcine lung model of IAH [10,13].

We used oleic acid to create lung injury because the resulting physiologic derangements mimic those of ALI in critically ill patients with interstitial edema, hemorrhagic and neutrophilic infiltration as well as air space edema and fibrin deposition resulting in impaired gas exchange (increase in ventilation/perfusion mismatching, intrapulmonary shunt, and dead space ventilation) [18].

Some of the results have previously been reported as an abstract [19].

## Materials and methods

The study conformed to the regulations of the Australian Code of Practice for the care and use of animals for scientific purposes and was approved by the Animal Ethics Committee of the University of Western Australia.

### Preparation of animals, anesthesia and ventilation

Nine male pigs (Large White breed) with a mean (SD) animal weight of 48 (6) kg were included in this study. Following an intramuscular sedation (tiletamine, zolazepam, and xylazine) anesthesia was maintained with propofol, morphine, and ketamine as previously described [10]. Neuromuscular blocking agents were not administered.

The pigs were mechanically ventilated (Evita2Dura, Draeger, Lübeck, Germany) via a size 8 endotracheal tube using the following settings: volume control (IPPV), FiO_2 _0.6, inspiration to expiration ratio = 1:1.5, inspiratory flow 40 L/minute, tidal volume 8 ml/kg with the initial respiratory rate adjusted to maintain an end tidal CO_2 _tension of 35 to 45 mmHg. With the exception of PEEP, the ventilation settings were not changed during the entire protocol. The initial PEEP setting was 5 cmH_2_O and was altered according to the experimental protocol.

### Respiratory mechanics and lung volumes

Esophageal pressure (P_ES_) was recorded using a thin-walled latex balloon (10-cm long) sealed over one end of a polyethylene catheter (Cardinal Health, Hoechberg, Germany) connected to a pressure transducer [13]. Following gastric insertion, the catheter was retracted stepwise until optimal position in the esophagus was confirmed with a positive occlusion test [20]. Airway pressure (P_AW_) was transduced at the proximal end of the endotracheal tube. End-inspiratory (_EI_) and end-expiratory (_EE_) pressures were obtained after a pause of three seconds. The static elastances were obtained by dividing the delta P_AW _(P_AW EI _- PEEP) for the respiratory system elastance (E_RS_) and the delta P_ES _(P_ES EI _- P_ES EE_) for the chest wall elastance (E_CW_) by the tidal volume. The static elastance of the lung (E_L_) was derived as E_L _= E_RS _- E_CW_. Transpulmonary pressures and transdiaphragmatic pressures were taken to be P_AW _- P_ES _and IAP - P_ES_, respectively. End-expiratory lung volume (EELV) was measured using the multiple breath nitrogen wash-out method [10,21]. Arterial oxygen tension (PaO_2_), oxygen saturation, carbon dioxide tension and hemoglobin concentration and mixed venous oxygen tension and oxygen saturation were measured with a blood gas analyzer immediately following collection (Rapidlab 1200, Siemens, Leverkusen, Germany). PaO_2 _over fractional inspiratory oxygen concentration (P/F ratio) was calculated. Shunt and dead-space fraction were calculated using standard formulae [22].

### Hemodynamic parameters

Mean arterial blood pressure (MAP) was measured at the femoral artery and cardiac output (CO) was measured by trans-cardiac thermodilution [10]. Throughout the study the animals remained supine. All hemodynamic pressures and IAP [23] were zeroed at the mid axillary line at the level of the sternum and measured during end-expiration. Powerlab and Labchart (ADI Instruments, Bella Vista, Australia) allowed continuous pressure measurement storage and *post-hoc *data analysis. Systemic vascular resistance (SVR) was calculated using a standard formula [10].

### Intra-abdominal pressure generation and measurement

A large bore orogastric tube was inserted to allow continuous gastric drainage. Different levels of IAP were generated using a large intra-abdominal latex balloon [13]. IAP was measured using a small latex balloon (as used to measure P_ES_) placed in the intra-abdominal cavity, below the liver. Abdominal perfusion pressure (APP) was calculated to be MAP - IAP [23].

### Acute lung injury

After a set of baseline measurements, oleic acid (Sigma-Aldrich, Steinheim, Germany) was given into the internal jugular vein to create ALI [18]. After an initial bolus of 0.04 ml/kg, a further bolus of 0.01 ml/kg was given every 10 minutes until a P/F ratio of 200 to 300 mmHg was established. Noradrenaline IV was used to maintain a MAP ≥70 mmHg during the infusion of oleic acid. Intravenous fluid administration was limited to 1 mL/kg/hour, after an initial 500 mL over a 30-minute bolus of succinylated gelatin.

### Experimental protocol

All nine pigs received oleic acid, two control pigs were instrumented without entering the experimental protocol to assess the stability of ALI; therefore, the investigation was conducted with seven pigs. Three different levels of IAP were randomly established either by not inflating (baseline IAP) or inflating the abdominal balloon with air to produce grade II (18 +/- 2 mmHg; 24.5 cmH_2_O) or grade III IAH (22 +/- 2 mmHg; 29.9 cmH_2_O) [23].

At each IAH setting, when initially applying baseline PEEP (5 cmH_2_O), norepinephrine was titrated until stable APP >70 mmHg was established. Thereafter, we did not change the norepinephrine infusion rate, in order to assess the hemodynamic effect of the different PEEP levels.

Different degrees of IAP-matching PEEP were randomly applied. At baseline IAP, baseline PEEP (5 cmH_2_O) and positive control PEEP (15 cmH_2_O) were applied. At grade II and III IAH, baseline PEEP (5 cmH_2_O), moderate PEEP (0.5 × IAP in cmH_2_O) and high PEEP (1.0 × IAP in cmH_2_O) were applied. The absolute levels of PEEP for each IAP level are given in Tables 1 and 2. For randomization, we used a split plot design [10].

**Table 1:** Respiratory effect of acute lung injury (ALI) and different levels of positive end-expiratory pressures (PEEP) at different levels of intra-abdominal pressures (IAP).

	Before ALI	After ALI							
PEEP cmH_2_O (mmHg)	Baseline	Baseline	High	Baseline	Moderate	High	Baseline	Moderate	High
	5 (3.7)	5 (3.7)	15 (11.0)	5 (3.7)	12 (8.8)	25 (18.4)	5 (3.7)	15 (11.0)	30 (21.1)
IAP cmH_2_O (mmHg)	Baseline	Baseline	Baseline	Grade II IAH	Grade II IAH	Grade II IAH	Grade III IAH	Grade III IAH	Grade III IAH
	5.9 (4)	5.9 (4)	5.9 (4)	24.5 (18)	24.5 (18)	24.5 (18)	29.9 (22)	29.9 (22)	29.9 (22)

PEEP measured, cmH_2_O	6 (0.2)	7 (0.3)	17 (0.4)	7 (0.2)	14 (0.2)	27 (0.3)	6 (0.4)	17 (0.3)	32 (0.3)
P_AW EI_, cmH_2_O	17 (1)	20 (4)^b^	32 (4)^c^	32 (6)^a^	37 (5)^d^	48 (3)^e,f^	34 (6)^a^	40 (4)^d^	54 (3)^e,f^
P_ES EE_, cmH_2_O	7 (3)	8 (2)	10 (2)^c^	8 (2)	11 (2)^d^	17 (6)^e,f^	8 (2)	13 (4)^d^	18 (7)^e,f^
P_ES __EI_, cmH_2_O	10 (2)	12 (2)	14 (3)	18 (6)^a^	20 (7)	24 (11)^f^	19 (6)^a^	21 (8)	23 (11)
P_TP __EE_, cmH_2_O	-1 (3)	-1 (2)	7 (2)^c^	-1 (2)	3 (3)^d^	10 (6)^e,f^	-2 (1)	4 (4)^d^	14 (7)^e,f^
P_TP EI_, cmH_2_O	7 (2)	8 (4)^b^	18 (6)^c^	15 (7)^a^	18 (9)	24 (12)^e,f^	16 (9)^a^	20 (10)	30 (13)^e,f^
P_TD EE_, cmH_2_O	0 (5)	2 (6)	0 (4)	17 (1)^a^	13 (3)^d^	8 (5)^e,f^	19 (3)^a^	13 (4)^d^	11 (7)^e^
P_TD EI_, cmH_2_O	-1 (4)	-1 (6)	-2 (4)	10 (4)^a^	9 (6)	10 (9)	10 (7)^a^	10 (7)	13 (11)
E_CW_, cmH_2_O/L	8 (2)	9 (3)	8 (4)	25 (10)^a^	19 (9)^d^	17 (11)^e,f^	29 (11)^a^	19 (12)^d^	14 (13)^e^
E_L_, cmH_2_O/L	20 (2)	23 (6)	27 (8)	37 (12)^a^	34 (12)	32 (10)	38 (14)^a^	34 (9)	37 (12)
PaCO_2_, mmHg	44 (2)	52 (4)^b^	50 (6)	60 (6)^a^	58 (6)	52 (5)^e,f^	59 (4)^a^	56 (4)	51 (8)^e,f^

^a^significant compared with baseline IAP; ^b^significant between before versus after ALI; ^c^significant between 5 versus 15 cmH_2_O; ^d^significant between baseline versus moderate PEEP; ^e^significant between baseline versus high PEEP; ^f^significant between moderate versus high PEEP. E_CW_, static elastance of chest wall; _EE_, end-expiratory; _EI_, end-inspiratory; E_L_, static elastance of lung; IAH, intra-abdominal hypertension; P_AW_, airway pressure; P_ES_, esophageal pressure; P_TD_, trans-diaphragmatic pressure (IAP - P_ES_); P_TP_, trans-pulmonary pressure (P_AW _- P_ES_); PaCO_2_, partial pressure of carbon dioxide. Mean (SD) are given. ANOVA and *post hoc *Student-Newman-Keuls were used for statistical testing (*P *<0.05). No significance analyzed for measured PEEP. ANOVA, analysis of variance.

**Table 2:** Hemodynamic effects of acute lung injury (ALI) and different levels of positive end-expiratory pressures (PEEP) at different levels of intra-abdominal pressures (IAP).

	Before ALI	After ALI							
PEEP cmH_2_O (mmHg)	Baseline	Baseline	High	Baseline	Moderate	High	Baseline	Moderate	High

	5 (3.7)	5 (3.7)	15 (11.0)	5 (3.7)	12 (8.8)	25 (18.4)	5 (3.7)	15 (11.0)	30 (21.1)

IAP cmH_2_O (mmHg)	Baseline	Baseline	Baseline	Grade II IAH	Grade II IAH	Grade II IAH	Grade III IAH	Grade III IAH	Grade III IAH

	5.9 (4)	5.9 (4)	5.9 (4)	24.5 (18)	24.5 (18)	24.5 (18)	29.9 (22)	29.9 (22)	29.9 (22)

MAP, mmHg	85 (13)	80 (10)	56 (14)^c^	85 (15)	75 (8)	56 (11)^e,f^	96 (8)	87 (19)	72 (19)^e^
APP, mmHg	81 (14)	73 (10)	49 (15)^c^	67 (15)	57 (7)	37 (12)^e,f^	77 (8)	69 (20)	52 (19)^e,f^
HR, beats/min	78 (17)	100 (14)	103 (21)	139 (46)^a^	139 (45)	138 (45)	117 (31)	112 (23)	134 (38)
CVP, mmHg	5 (4)	5 (3)	9 (3)^c^	6 (1)	11 (5)^d^	17 (3)^e,f^	5 (2)	13 (2)^d^	18 (2)^e,f^
PAP, mmHg	15 (3)	35 (7)^b^	33 (6)	39 (9)^a^	38 (9)	37 (7)	41 (10)^a^	41 (8)	43 (7)
SVR, dyn * s/ cm^5^	1506 (210)	1546 (215)	1450 (355)	1822 (233)	1783 (506)	1771 (355)	2091 (323)^a^	2049 (275)	1996 (540)

^a^significant compared with baseline IAP; ^b^significant between before versus after ALI; ^c^significant between 5 versus 15 cmH_2_O; ^d^significant between baseline versus moderate PEEP; ^e^significant between baseline versus high PEEP; ^f^significant between moderate versus high PEEP. APP, abdominal perfusion pressure; CVP, central venous pressure; HR, heart rate; IAH, intra-abdominal hypertension; MAP, mean arterial pressure; PAP, mean pulmonary artery pressure; SVR, systemic vascular resistance. Mean (SD) are given. ANOVA and *post hoc *Student-Newman-Keuls were used for statistical testing (*P *<0.05). No significance analyzed for measured PEEP. ANOVA, analysis of variance.

A standardized lung recruitment maneuver was performed by applying 40 cmH_2_O for 30 seconds after each new PEEP level was set [17]. All measurements were performed after a 10 minute stabilization period.

### Statistics

Previous sample size calculations showed that seven subjects were sufficient to identify a difference in P/F ratio of 50 mmHg (assuming a mean (SD) P/F ratio of 120 (45) mmHg) between two different PEEP values (α = 0.05, power = 80%). Data are reported as mean (SD), as the data proved to be normally distributed, when analyzed by the Kolmogorov-Smirnov test. To compare the data between the different combinations of PEEP and IAP, an analysis of variance (ANOVA) for repeated measures was performed and a *post hoc *Student-Newman-Keuls-test was used to adjust for multiple comparisons. A probability of <0.05 was considered statistically significant.

## Results

At baseline, IAP was 5.9 (2.3) cmH_2_O. No pneumothorax was observed in any subject.

### Cardio-respiratory effect of oleic acid

**Figure 1:**
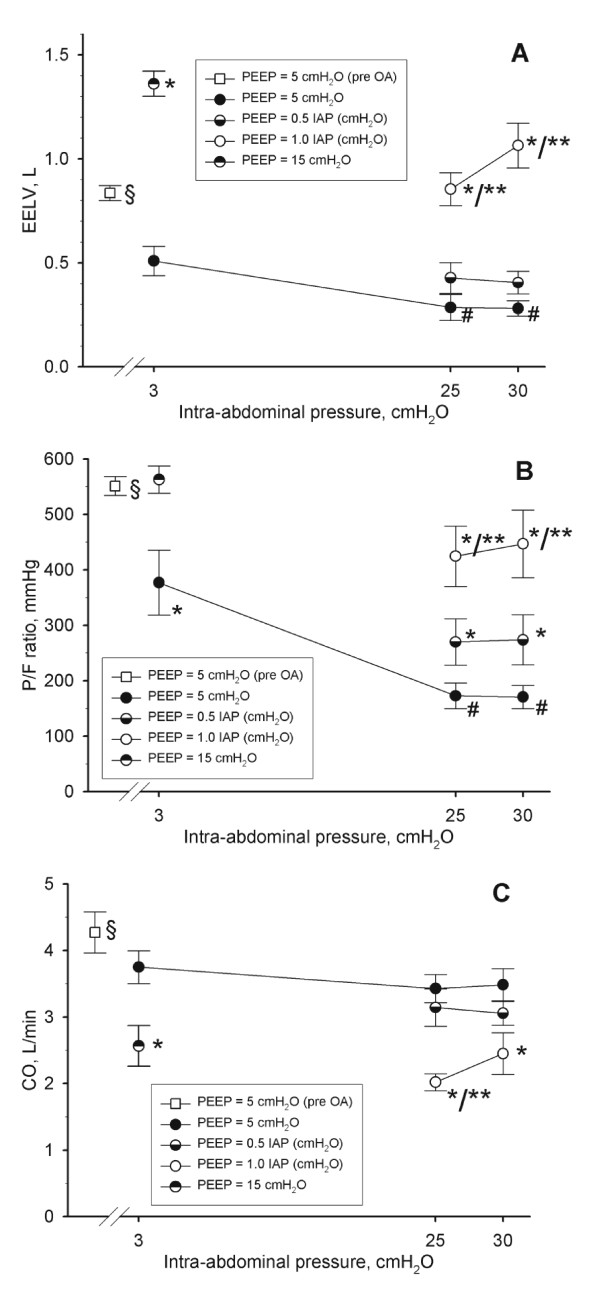
**End-expiratory lung volumes in L (**A**), arterial oxygen tension/fractional inspiratory concentration of oxygen (P/F ratio) in mmHg (**B**) and cardiac output (CO) in L/minute (**C**) in function of different levels of intra-abdominal hypertension (IAH) (baseline (3 cmH_2_O), grade II IAH, (25 cmH_2_O = 18 mmHg), and grade III IAH (30 cmH_2_O = 22 mmHg)) at different degrees of IAP-matching levels of positive end-expiratory pressures (PEEP)**. Mean and SE are shown. ANOVA and *post hoc *Student-Newman-Keuls were used for statistical testing. §, *P *<0.05 comparing before and after oleic acid (baseline IAP and 5 cmH_2_O PEEP). *, *P *<0.05 within an IAP setting versus the corresponding value at 5 cmH_2_O PEEP. **, *P *<0.05 within an IAP setting comparing moderate versus high PEEP. #, *P *<0.05 within a PEEP setting versus the corresponding value at baseline IAP. ANOVA, analysis of variance.

**Figure 2:**
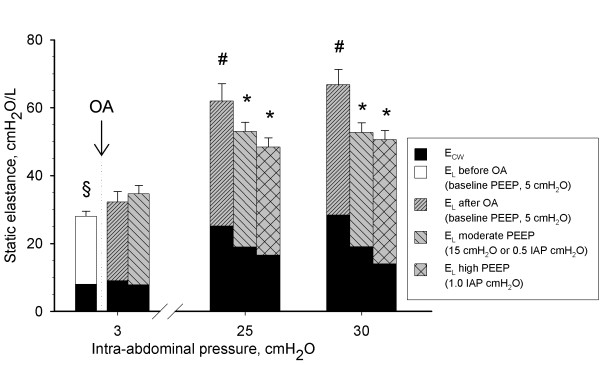
**Static elastance of the total respiratory system (E_RS_) as a composite of chest wall (E_CW_, black filled bar) and lung elastance (E_L_, white or grey filled bar) in function of different levels of intra-abdominal hypertension (IAH) (baseline (3 cmH_2_O), grade II IAH, (25 cmH_2_O = 18 mmHg), and grade III IAH (30 cmH_2_O = 22 mmHg)) at different degrees of IAP-matching levels of positive end-expiratory pressures (PEEP)**. OA, oleic acid. Mean and SE (E_RS_) are shown. ANOVA and *post hoc *Student-Newman-Keuls were used for statistical testing. §, *P *<0.05 comparing E_RS _before and after oleic acid. *, *P *<0.05 E_RS _within an IAP setting versus the corresponding value at 5 cmH_2_O PEEP. There were no significant differences in E_RS _within an IAP setting comparing moderate versus high PEEP. #, *P *<0.05 E_RS _within a PEEP setting versus the corresponding value at baseline IAP. ANOVA, analysis of variance.

To generate ALI (P/F ratio 200 to 300 mmHg) a mean cumulative dose of 0.30 (0.41) ml/kg oleic acid was given IV. Thirty minutes after ALI was established, 0.2 (0.4) mcg/minute of IV norepinephrine was required to maintain an APP >70 mmHg. Oleic acid decreased the P/F ratio, EELV, and CO, and increased PAP and E_RS _(Tables 1 and 2, Figures 1 and 2).

After the generation of ALI, the measured parameters including the P/F ratio, EELV, CO, and MAP remained stable over four hours in the two control animals (data not shown). The remaining seven animals completed the experimental protocol each within four hours.

### Respiratory effect of IAP and PEEP

**Figure 3:**
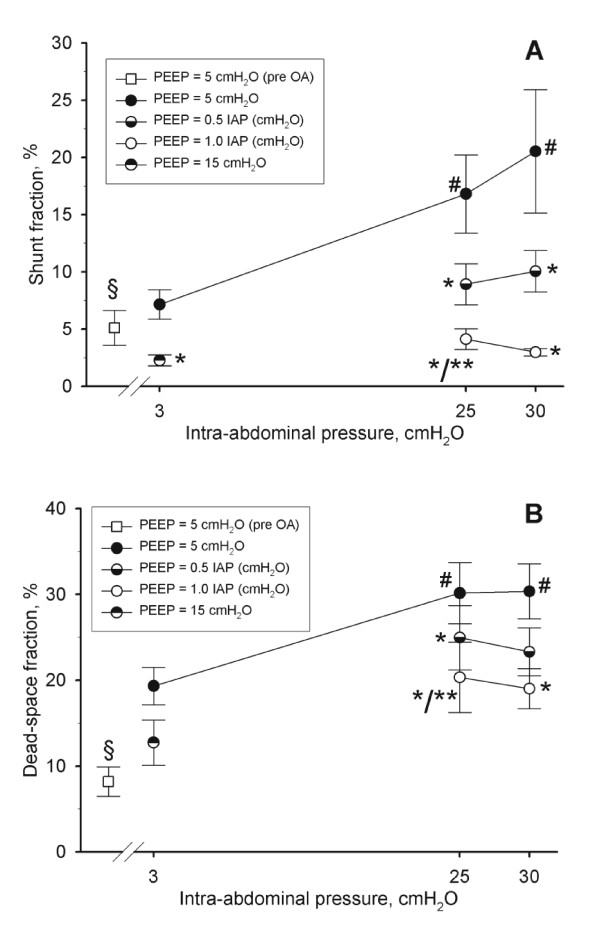
**Shunt fraction in % (**A**) and dead-space fraction in % (**B**) in function of different levels of intra-abdominal hypertension (IAH) (baseline (3 cmH_2_O), grade II IAH (25 cmH_2_O = 18 mmHg), and grade III IAH (30 cmH_2_O = 22 mmHg)) at different degrees of IAP-matching levels of positive end-expiratory pressures (PEEP)**. Mean and SE are shown. ANOVA and *post hoc *Student-Newman-Keuls were used for statistical testing. §, *P *<0.05 comparing before and after oleic acid (baseline IAP and 5 cmH_2_O PEEP). *, *P *<0.05 within an IAP setting versus the corresponding value at 5 cmH_2_O PEEP. **, *P *<0.05 within an IAP setting comparing moderate versus high PEEP. #, *P *<0.05 within a PEEP setting versus the corresponding value at baseline IAP. ANOVA, analysis of variance.

Grade II and III IAH further decreased EELV and the P/F ratio, whereas PEEP reversed this in a dose related manner (Figure 1). The changes in EELV paralleled those seen in the P/F ratio (Figure 1). Shunt and dead-space fraction decreased with increasing IAH and decreased with increasing PEEP (Figure 3).

EELV, P/F ratio, and E_CW _correlated better with end-expiratory IAP-PEEP than with end-expiratory transdiaphragmatic pressure or transpulmonary pressure whereas E_RS _and E_L _correlated better with end-expiratory transdiaphragmatic pressure than with end-expiratory transpulmonary pressure or IAP-PEEP [see Additional file 1].

### Respiratory mechanics

At baseline PEEP, IAH increased E_RS_, E_CW _and E_L _(Figure 2). At baseline IAP, PEEP did not increase E_RS _significantly. In the presence of IAH, PEEP decreased E_RS _by decreasing E_CW_.

At baseline PEEP, IAH increased end-inspiratory transpulmonary pressures but did not influence end-expiratory transpulmonary pressures. PEEP caused a dose related increase in end-inspiratory and end-expiratory transpulmonary pressure.

### Cardiac effect of IAP and PEEP

To maintain an APP >70 mmHg, 0.02 (0.03), 0.07 (0.08), and 0.06 (0.07) mcg/kg/minute of norepinephrine IV was required at baseline IAP, grade II, and grade III IAH, respectively. PEEP was associated with a dose-related decrease in CO and MAP at all grades of IAH (Table 2 and Figure 1). PEEP had no effect on systemic vascular resistance.

## Discussion

In this porcine sick lung model with IAH, we examined the effect of IAP-matching PEEP on cardio-respiratory parameters. Our main findings were that, in the presence of ALI, IAH (grade II and III) reduced EELV and the P/F ratio and increased shunt and dead-space fraction as well as E_RS _by increasing both E_CW _and E_L_. PEEP increased EELV and the P/F ratio, in a dose-dependent manner and when fully matched with IAP, abolished the IAH-induced declines in EELV and the P/F ratio. IAP-matching PEEP reduced shunt and dead-space fraction as well as E_RS _due to a reduction in E_CW_. Furthermore, high IAP-matching PEEP caused a reduction in CO.

### Effect of IAH on respiratory function

In this study, in the presence of ALI, IAH caused a parallel decrease in EELV and the P/F ratio that can be explained by an increase in shunt and dead-space fraction. Furthermore, in keeping with the literature, we found IAH to increase E_RS _due to an increase in E_L _and E_CW _[8].

These findings in injured lungs contrast with previous observations found in healthy lungs in comparable animal models, where IAH increased E_RS _by increasing predominantly E_CW _[8].

### Effect of oleic acid on respiratory function

Consistent with the literature, we found that oleic acid decreased EELV, oxygenation and CO and increased shunt and dead-space fraction and PAP [8,18]. Although we did not find any differences in respiratory mechanics, probably due to the small sample size, oleic acid has been reported to increase E_RS _and E_L _without any effect on E_CW _[8].

### The effect of PEEP in the presence of ALI and IAH

In this porcine sick lung model, IAP-matching PEEP not only increased EELV but also improved gas exchange due to a reduced shunt and dead-space fraction. Furthermore, PEEP in this setting decreased E_RS _by decreasing E_CW _with no effect on E_L_.

In a previous study, Gattinoni *et al. *found that in patients with pulmonary ARDS (n = 12), increasing PEEP (up to 15 cmH_2_O) increased E_RS _by increasing E_L _whereas in patients with extra-pulmonary ARDS (n = 9), PEEP decreased E_RS _by decreasing E_CW _and E_L_. However, the patients with extrapulmonary ARDS had IAH (mean IAP = 22 mmHg) [12] and it might well be that it was the presence of IAH and not the nature of ARDS that determined how PEEP affected respiratory mechanics and, ultimately, lung volumes and gas exchange. Unfortunately oxygenation was not assessed.

Krebs *et al. *also applied different PEEP levels (up to 20 cmH_2_O) in 20 patients with ARDS of which half did and half did not have IAH (mean IAP were 16 and 8 mmHg, respectively) [17]. PEEP was found to improve oxygenation and to decrease E_RS _by decreasing E_L _without influencing E_CW _in both groups. Lung recruitment volumes, but not residual lung volumes, were assessed.

The most likely reason why Krebs *et al. *did not find PEEP to influence E_CW _in patients with IAH and ARDS is that IAH did not affect respiratory mechanics in their patients. For example IAP neither influenced P_ES _nor consequently affected E_CW _in contrast to current and previous experimental [8,13] and clinical findings [11,12].

In general, IAH appears to decrease EELV and increase E_CW _independent of the condition of the lung and we attribute this to displacement of the diaphragm into the thorax and an increase in transdiaphragmatic pressure [8,12] whereas PEEP in the presence of IAH counteracts this effect and, thereby, increases EELV and decreases E_CW _also independent of an underlying lung injury [12].

Although we did not find PEEP to affect E_L _in the presence of IAH and lung injury, others have found E_L _to decrease in experimental [8,15] and clinical studies [12].

It is possible that E_L _did not change with PEEP in this study because the PEEP-induced decrease in atelectasis and recruitment of pulmonary units (causing a reduction in E_L_) [12] was counter-balanced by overdistension of non-dependent alveolar units (causing an increase in E_L_) [24] and might explain why we previously found an increase in E_L _caused by IAP-matching PEEP in the absence of ALI [13].

### Clinical consequences

What relevance do our experimental findings have for the critically ill patient with IAH? Although PEEP can improve lung volumes in patients with IAH and may appear beneficial, the potential benefits have to be weighed against the potential side effects when applying high levels of PEEP in such patients:

1) PEEP appears to improve oxygenation only in the setting of injured lungs and not in healthy lungs [10,13,15]. The effect of PEEP on improving oxygenation is probably independent of IAH as alveolar recruitablilty depends largely on the degree and distribution of underlying lung injury with the success rate being greatest in ARDS patients with low oxygen levels [22].

2) Although PEEP can reduce E_CW _and thereby counteract the effect of IAH [13], even when applying protective tidal volumes and maintaining a constant driving pressure, increasing PEEP will inherently increase inspiratory airway pressure. Generally, it is recommended that airway pressures should not exceed 30 cmH_2_O (which was exceeded in this study when applying high PEEP). In the context of IAH, limiting end-inspiratory transpulmonary pressures below 25 cmH_2_O has been the suggested method to avoid excessive alveolar overdistension as it is thought that not the actual airway but rather the resulting transpulmonary pressures (stress) are responsible for causing alveolar overdistension (strain) [25,26]. However, this requires the clinician to place an esophageal balloon catheter to estimate and calculate pleural and transpulmonary pressures, respectively. In support of using esophageal balloon catheters, a randomized controlled trial showed better oxygenation and a trend towards an improved outcome when targeting end-expiratory transpulmonary pressure in patients with ARDS [27].

Whether PEEP has a role in preventing ventilator-induced lung injury in the setting of IAH by preventing repeated opening and closing of recruitable lung units [28], remains to be investigated.

3) PEEP increases the risk of hemodynamic impairment [6,13]. For example, we found that high IAP-matching PEEP decreased CO. These values are comparable to our previous findings in a porcine model without ALI [13].

4) Furthermore, increased PEEP levels have been shown not only to promote fluid leakage by increasing venous and capillary pressures but also to impair abdominal and thoracic lymph drainage by compressing the thoracic lymph duct [29]. Any PEEP-induced improvement in oxygenation might, therefore, be offset by a worsening of IAH.

### Limitations

Several limitations have to be mentioned apart from the study being experimental, thereby limiting generalization of the results. 1) Neuromuscular blocking agents were not applied in accordance with our routine clinical practice. The potential of respiratory muscle activity influencing results cannot be excluded although we did not observe any monitored respiratory muscle activity during data analysis. 2) We applied tidal volumes of 8 ml/kg, which are higher than currently recommended for mechanical ventilation of patients with ALI/ARDS and have the potential to cause or further exacerbate ventilator induced lung injury (VILI) [25,30]. 3) As we designed this study to examine the cardio-respiratory effect of PEEP in the setting of IAH (and not that of IAH), we adjusted the initial noradrenaline rate between the investigated IAH levels (when applying PEEP of 5 cmH_2_O) but did not change the noradrenaline rate thereafter when applying different PEEP levels. A better approach might have been to adjust the noradrenaline concentration to achieve an APP >60 mmHg at all IAP and PEEP settings, thereby, allowing deductions to be made from the changes in noradrenaline rates.

## Conclusions

In conclusion, in this porcine sick lung model, IAP-matching PEEP decreased CO, shunt, dead space ventilation, and chest wall elastance, and increased lung volumes as well as oxygenation. The study shows that the effect of PEEP on oxygenation and respiratory mechanics in the setting of IAH depends on the underlying lung injury.

Our findings support the application of positive end-expiratory pressure that is adjusted to the degree of IAP in the setting of ALI and IAH. However, the potential benefit of improving oxygenation has to be weighed against potential alveolar overdistension with the potential to cause VILI and hemodynamic compromise. Moderate IAP-matching PEEP (0.5 × IAP) provided a reasonable balance between improved oxygenation and increased risk of hemodynamic compromise and alveolar overdistension. We encourage the use of an esophageal balloon catheter when applying higher PEEP levels in patients with respiratory compromise and IAH to avoid inspiratory transpulmonary pressures above 25 cmH_2_O.

Whether IAP-matching PEEP can protect against IAH-induced organ damage or can prevent cyclic opening and collapsing of alveoli and, thereby, reduce the risk of ventilator-associated lung injury remains to be investigated. We strongly encourage future research in the field of ALI and ARDS to consider the influence of IAH in the clinical setting when assessing how PEEP affects oxygenation, lung volumes and lung mechanics.

## Key messages

• In subjects with IAH but with healthy lungs, PEEP increases lung volumes but does not influence chest wall elastance or oxygenation.

• In this porcine model with sick lungs and IAH, PEEP increased lung volumes, oxygenation and decreased chest wall elastance.

• This study shows that lung injury and IAH modify how PEEP influences oxygenation and respiratory mechanics.

• To improve oxygenation, our findings support the application of PEEP in the setting of acute lung injury and IAH.

• However, high PEEP is also associated with alveolar overdistension and reduced cardiac output.

## Abbreviations

APP: abdominal perfusion pressure; ARDS: acute respiratory distress syndrome; ALI: acute lung injury; CO: cardiac output; _EI_: end-inspiratory; _EE_: end-expiratory; E_RS_: static elastance of the respiratory system; E_CW_: static elastance of the chest wall; E_L_: static elastance of the lung; EELV: end-expiratory lung volume; IAH: intra-abdominal hypertension; IAP: intra-abdominal pressure; IV: intravenous; MAP: mean arterial pressure; P_AW_: airway pressure; P_ES_: esophageal pressure; P/F ratio: arterial oxygen tension over fraction of inspiratory oxygen; PAP: pulmonary artery pressure; PEEP: positive end-expiratory pressure; SVR: systemic vascular resistance.

## Competing interests

The authors declare that they have no competing interests.

## Authors' contributions

AR, RM, and PVH participated in the conception, hypothesis delineation, and design of the study. AR, RM, EF, BR, and BN contributed to data acquisition. AR performed data interpretation and statistical analyses. AR drafted the manuscript. RM, EF, BR, BDK, BS, and PVH revised the manuscript. All authors read and approved the final manuscript.

## Supplementary Material

Additional file 1 **Various respiratory scatter plots**. Scatter plots depicting end-expiratory lung volume (EELV), arterial partial pressure per inspiratory fraction of oxygen (P/F ratio), static elastance of the respiratory system (E_RS_), of the chest wall (E_CW_) and of the lung (E_L_) against resulting end-expiratory transdiaphragmatic pressure, transpulmonary pressure and intra-abdominal pressure minus positive end-expiratory pressure (IAP-PEEP). Click here for file
