# Supplementary material for: Matching positive end-expiratory pressure to intra-abdominal pressure improves oxygenation in a porcine sick lung model of intra-abdominal hypertension
Source: Crit Care. 2012 Oct 26;16(5):R208. doi: 10.1186/cc11840 (PMC3682312; doi:10.1186/cc11840)
Supplement: Additional file 1 — Various respiratory scatter plots. Scatter plots depicting end-expiratory lung volume (EELV), arterial partial pressure per inspiratory fraction of oxygen (P/F ratio), static elastance of the respiratory system (ERS), of the chest wall (ECW) and of the lung (EL) against resulting end-expiratory transdiaphragmatic pressure, transpulmonary pressure and intra-abdominal pressure minus positive end-expiratory pressure (IAP-PEEP). [file cc11840-S1.DOC]

**Additional file 1:** **Various respiratory scatter plots**

Scatter plots depicting end-expiratory lung volume (EELV), arterial partial pressure per inspiratory fraction of oxygen (P/F ratio), static elastance of the respiratory system (E_RS_), of the chest wall (E_CW_) and of the lung (E_L_) against resulting end-expiratory transdiaphragmatic pressure, transpulmonary pressure and intra-abdominal pressure minus positive end-expiratory pressure (IAP-PEEP). PEEP was set in relation to IAP (see manuscript).
